# P-80. National Mortality Trends in Diabetes and its Complications, 1999-2020: Rising Impact of Osteomyelitis and Racial Disparities

**DOI:** 10.1093/ofid/ofaf695.309

**Published:** 2026-01-11

**Authors:** Andrew Wang, Krishna Sanaka, Marcos Schechter

**Affiliations:** Geisel School of Medicine at Dartmouth, Lebanon, NH; University of Pennsylvania, Cleveland, OH; Emory University School of Medicine, Atlanta, GA

## Abstract

**Background:**

Diabetes mellitus is a leading cause of morbidity in the United States, but mortality rates from its most severe complications—stroke, heart failure, hyperglycemic crisis, and osteomyelitis—remain undercharacterized. We analyzed national mortality trends associated with diabetes and major complications, focusing on osteomyelitis and related disparities.

**Figure:**
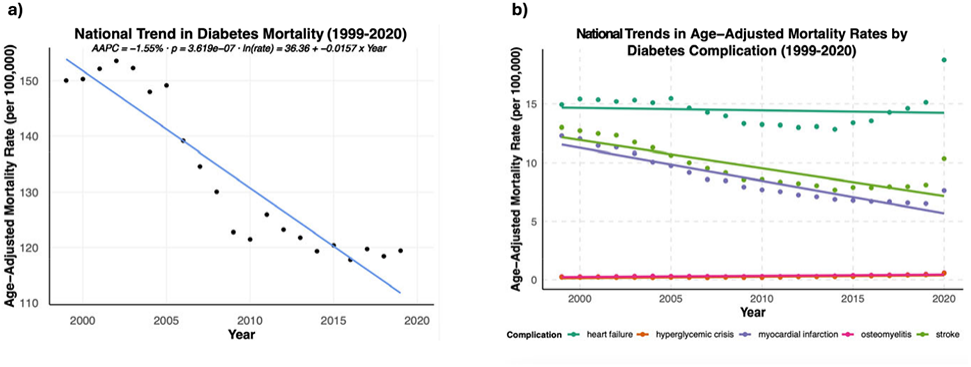
Trends in Diabetes-Related Mortality and Complication-Specific Mortality in the United States, 1999–2020 a, National diabetes-related AAMRs declined significantly from 1999–2020 (AAPC = –1.55%, p < 0.001). b, AAMRs by diabetes-related complication; osteomyelitis mortality increased modestly, diverging from declining trends in other complications.

**Figure:**
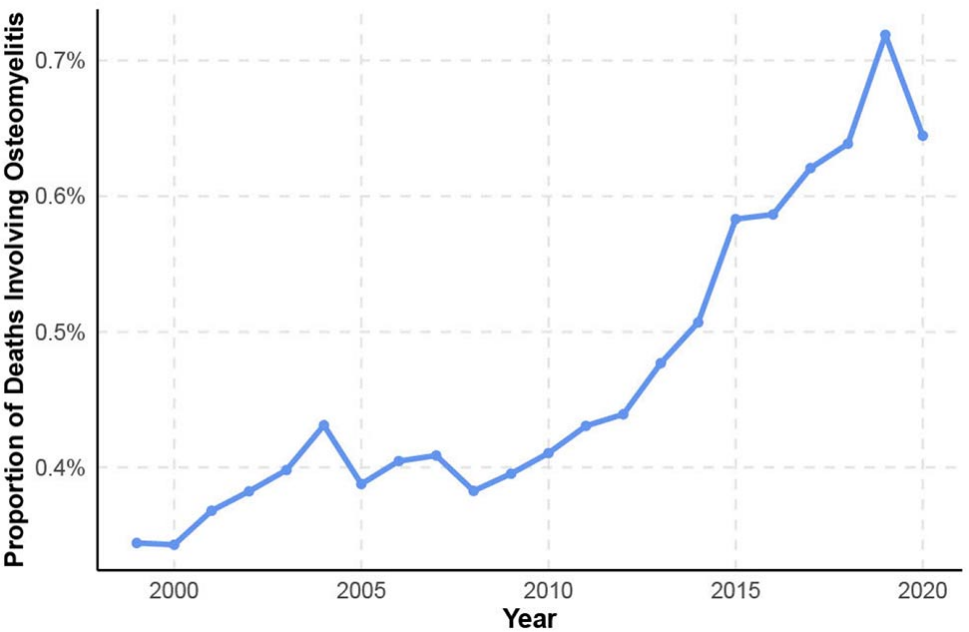
Rising Proportional Mortality Involving Osteomyelitis Among U.S. Diabetes Deaths, 1999–2020 Proportion of diabetes-related deaths involving osteomyelitis increased significantly from 1999–2020 (p < 0.0001, Cochran-Armitage test), highlighting a rising infectious contribution to diabetes mortality.

**Methods:**

We used CDC WONDER Multiple Cause-of-Death data (1999-2020) to identify U.S. deaths listing diabetes and selected complications (ESRD, hyperglycemic crisis, heart failure, stroke, myocardial infarction, and osteomyelitis) via ICD-10 codes. We calculated age-adjusted mortality rates (AAMRs) by sex and race/ethnicity, and assessed temporal trends by Average Annual Percent Change (AAPC), derived from joinpoint regression models to estimate average change in mortality over time. For diabetes with osteomyelitis, analyses included proportional mortality, temporal trends, and mortality rate ratios (MRRs).

**Figure:**
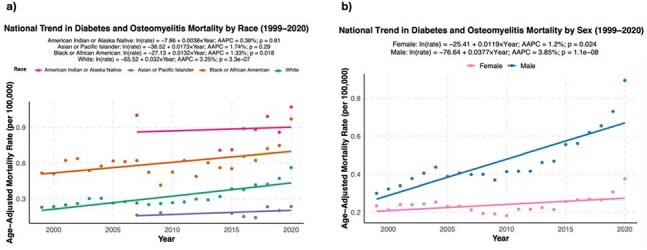
Age-Adjusted Mortality Trends for Comorbid Diabetes and Osteomyelitis by Race and Sex, United States, 1999–2020 a, Scatter plots and fitted log-linear regression lines showing trends in age-adjusted mortality rates (AAMRs) for diabetes-related deaths involving osteomyelitis, stratified by race. Black individuals experienced a persistently high and significantly rising mortality burden, highlighting sustained disparities over time. White individuals had the steepest increase, while American Indian or Alaska Native individuals had the highest AAMRs but no statistically significant trend. b, Scatter plots and fitted regression lines illustrating trends in AAMRs by sex. Male individuals experienced consistently higher mortality rates than females, with a significantly steeper increase over time.

**Figure:**
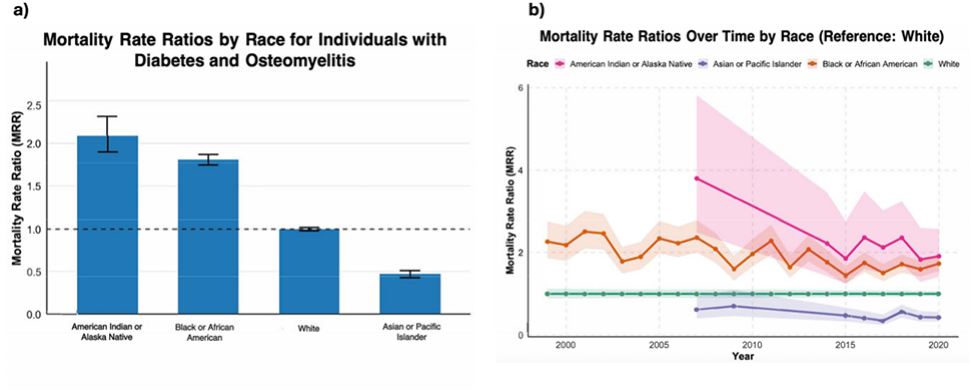
Racial Disparities in Mortality Rate Ratios for Comorbid Diabetes and Osteomyelitis: National Cross-Sectional and Temporal Trends, 1999–2020 a, Bar graph showing overall MRRs by race for individuals with comorbid diabetes and osteomyelitis, using White individuals as the reference group (MRR = 1.00). Black or African American individuals had an MRR of 1.81 (95% CI: 1.75–1.87), and American Indian or Alaska Native individuals had the highest overall MRR at 2.09 (95% CI: 1.90–2.32). Asian or Pacific Islander individuals had significantly lower mortality (MRR = 0.47; 95% CI: 0.43–0.51). b, Line graph showing temporal trends in MRRs by race from 1999 to 2020, relative to White individuals (MRR = 1.00 annually). Black and American Indian or Alaska Native populations consistently exhibited elevated MRRs, though variability and wider confidence intervals were noted for the latter. Asian or Pacific Islander individuals maintained lower and relatively stable MRRs over the study period.

**Results:**

Diabetes-related deaths decreased significantly in the US from 1999-2020 (AAPC = -1.33%, p < .01) (Figure 1a). While the AAMR for most diabetes complications decreased significantly, the AAMR for hyperglycemic crisis (AAPC = 4.03%, p < .01) and osteomyelitis (AAPC = 2.9%, p < .01) increased significantly (Figure 1b). Among 1,674,724 diabetes-related deaths, 26,022 (1.55%) involved osteomyelitis, and the proportion of diabetes-related deaths involving osteomyelitis increased from 0.34% to 0.64% during the study period (Figure 2). Among underrepresented racial and ethnic groups, Black persons experienced the sharpest increase in osteomyelitis-associated mortality, with males also showing an upward trend (Figure 3). Black and American Indian/Alaska Native persons had higher MRR than white persons, with disparities persisting over time (Figure 4).

**Conclusion:**

Mortality involving comorbid diabetes and osteomyelitis has increased markedly, with disproportionately high and rising burdens among Black and American Indian/Alaska Native populations. These findings identify osteomyelitis as an emerging infectious contributor to diabetes-related mortality and emphasize the need for equity-driven, preventive interventions.

**Disclosures:**

All Authors: No reported disclosures

